# A novel classification of posterior pelvic exenteration to assess prognosis in female patients with locally advanced primary rectal cancer: a retrospective cohort study from China PelvEx collaborative

**DOI:** 10.1007/s00384-024-04632-9

**Published:** 2024-04-26

**Authors:** Yuegang Li, Meng Zhuang, Gang Hu, Jinzhu Zhang, Wenlong Qiu, Shiwen Mei, Jianqiang Tang

**Affiliations:** Department of Colorectal Surgery, National Clinical Research Center for Cancer/Cancer Hospital, National Cancer Center, Chinese Academy of Medical Sciences and Peking Union Medical College, Beijing, 100021 China

**Keywords:** Posterior pelvic exenteration, Oncological outcome, Peking classification, Locally advanced rectal cancer

## Abstract

**Purpose:**

Surgical techniques and the prognosis of posterior pelvic exenteration for locally advanced primary rectal cancer in female patients pose challenges that need to be addressed. Therefore, we investigated the short-term and survival outcomes of posterior pelvic exenteration in female patients using a novel Peking classification.

**Methods:**

We retrospectively analysed a prospective database from China PelvEx Collaborative across three tertiary referral centres. A total of 172 patients who underwent combined resection for locally advanced primary rectal cancer were classified based on four subtypes (PPE-I [64/172], PPE-II [68/172], PPE-III [21/172], and PPE-IV [19/172]) according to the Peking classification; perioperative characteristics and short-term and oncological outcomes were analysed.

**Results:**

Differences were significant among the four groups regarding colorectal reconstruction (*p* < 0.001), perineal reconstruction (*p* < 0.001), in-hospital complications (*p* < 0.05), and urinary retention (*p* < 0.05). The R_0_ resection rates for PPE-I, PPE-II, PPE-III, and PPE-IV were 90.6%, 89.7%, 90.5%, and 89.5%, respectively. The 5-year overall survival rates of the PPE-I, PPE-II, PPE-III, and PPE-IV groups were 73.4%, 68.8%, 54.7%, and 37.3%, respectively. Correspondingly, their 5-year disease-free survival rates were 76.0%, 62.5%, 57.7%, and 43.1%, respectively. Notably, the PPE-IV group demonstrated the lowest 5-year overall survival rate (*p* < 0.001) and 5-year disease-free survival rate (*p* < 0.001).

**Conclusion:**

The Peking classification can aid in determining suitable surgical techniques and conducting prognostic assessments in female patients with locally advanced primary rectal cancer.

**Supplementary Information:**

The online version contains supplementary material available at 10.1007/s00384-024-04632-9.

## Introduction

Locally advanced rectal cancer (LARC) often requires neoadjuvant chemoradiation (nCRT) combined with total mesorectal excision to achieve complete resection margins, minimise local recurrence rates, and enhance survival. While the European Society for Medical Oncology guidelines endorse nCRT for LARC [1], the debate over its ability to achieve satisfactory tumour stage reduction and local control in LARC remains controversial and requires further clarification [2]. Extensive surgery, which is commonly the only recourse for achieving complete resection for local control and palliation, is associated with insufficient patient survival rates. Total pelvic exenteration (TPE), involving resection of the rectum, bladder, lower ureters, and internal reproductive organs, may impact patient survival [3]. However, TPE is an invasive radical treatment approach and is associated with considerable morbidity and mortality [4, 5].

Additionally, TPE is relatively rare in female patients owing to their anatomy. Conversely, posterior pelvic exenteration (PPE), involving resection of the uterus, cervix, and vagina, is a more commonly performed procedure. Despite the growing incidence of PPE procedures in the context of gynaecological tumours [6], the utilisation of PPE procedures for rectal cancer remains limited, and there are only a handful of reports, which have small sample case descriptions [7]. The use of PPE in the treatment of rectal cancer has not been extensively studied, particularly concerning surgical methods and oncological outcomes. Moreover, performing PPE for rectal cancer presents more challenges compared to TPE due to the imperative of organ preservation while achieving R_0_ resection [8]. Consequently, a dedicated study on LARC involving internal reproductive organs and evaluating both short-term and oncological outcomes is required.

In this study of a specific group of individuals, carried out through the China PelvEx collaboration, we utilised a novel classification system for PPE and comprehensively investigated surgical outcomes and oncological prognosis based on this classification. Within this classification, PPE was categorised into four subtypes: PPE-I (uterine invasion), PPE-II (upper vaginal invasion), PPE-III (lower vaginal invasion), and PPE-IV (major vaginal invasion). As this new classification is based on TNM staging and rectal MRI, its use might better inform future clinical decisions.

## Materials and methods

### Ethical approval and patient consent

This study received approval from the ethics committees of several institutions, including the Cancer Hospital of the Chinese Academy of Medical Sciences, Peking University First Hospital, and Gansu Provincial Hospital (approval number 22/442–3644). Written consent was obtained from all participants.

### Study design

This study follows the STROBE reporting guidelines. This retrospective study employed a multicentre approach to analyse data derived from a prospectively compiled institutional database and tumour registry. This study focused on cT4b female patients who underwent multivisceral resection (MVR) from three institutions in the China PelvEx Collaboration between January 2010 and December 2020. The study included consecutive female patients with LARC who underwent PPE. The inclusion criteria were: (1) female patients with LARC (cT4b), (2) radical resection, and (3) histologically confirmed adenocarcinoma. The exclusion criteria were: (1) total pelvic exenteration (TPE), (2) recurrent rectal cancer, (3) simultaneous distant metastasis, (4) ovarian or adnexal proctectomy, and (5) PPE with sacrectomy.

Prior to surgery, each patient underwent a comprehensive preoperative multidisciplinary consultation, wherein experts from various fields, including radiotherapy, colorectal surgery, urology, gynaecology, and plastic surgery, were brought together. The salient role of multidisciplinary team meetings was underscored in the formulation of personalised treatment strategies. These deliberations encompassed pivotal decisions, including the selection of surgical approach (laparoscopic or open) and consideration of the potential use of preoperative chemoradiotherapy.

### Preoperative management

For those in the clinical stage of T4b, nCRT was advised, involving a standardised long-course chemotherapy regimen centred on 5-fluorouracil (5-FU), concomitant with external radiation administered at a cumulative dose of 45–54 Gy. Subsequent to nCRT, patients underwent reassessment through pelvic MRI. Surgery was scheduled approximately 8–12 weeks after completing the nCRT.

### Data collection and follow-up

Information on a range of clinicopathological factors, including age, body mass index (BMI), American Society of Anesthesiologists (ASA) score, neoadjuvant treatment, tumour size, and pathologic stage, as well as serum levels of haemoglobin (Hb), albumin (Alb), carcinoembryonic antigen (CEA), and carbohydrate antigen 199 (CA199) was collected. Additionally, perioperative outcomes were documented, including operative time, blood loss, type of colorectal and perineal reconstruction, circumferential resection margin (CRM) status, the number of retrieved or positive lymph nodes, morbidity, length of hospital stay, and postoperative complications. The classification of complications followed the Clavien–Dindo system, which differentiates between minor (grades I or II) and major surgical complications (grades III, IV, or V). Patient follow-up took place through telephone conversations or outpatient department visits. The primary endpoints of this study were 5-year overall survival (OS), disease-free survival (DFS), and 5-year local recurrence survival (LRS) in alignment with the Peking classification. Additionally, secondary endpoints were directed towards the assessment of surgical complications using the Peking classification and the exploration of prognostic factors influencing survival outcomes.

### New classification and surgical procedures

**Fig. 1:**
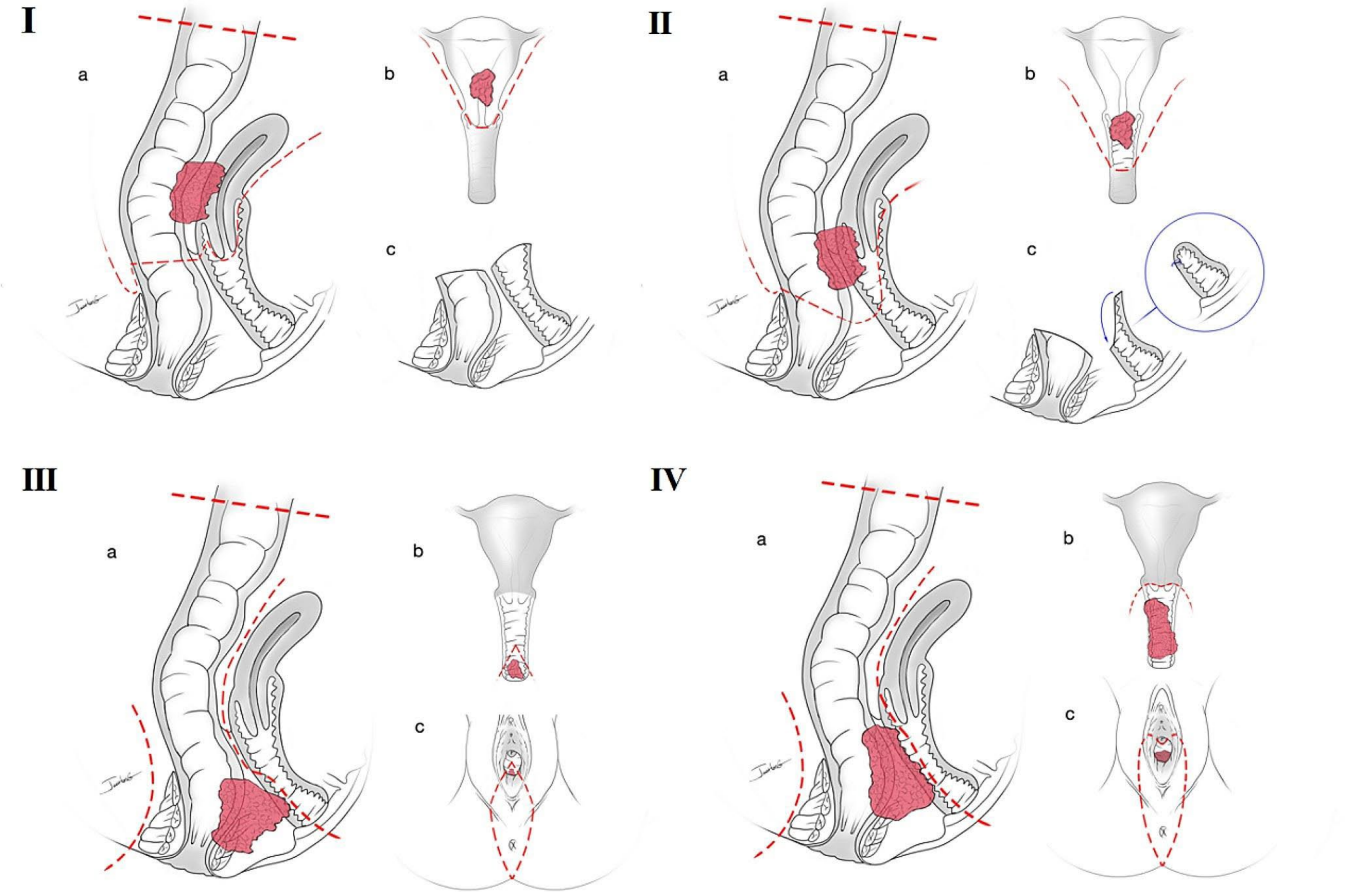
Schematic of the Peking classification of PPE

The absence of a universally agreed-upon definition for PPE within rectal cancer surgery has led to various interpretations. Some definitions have been influenced by those established for gynaecological tumours [9, 10], while others emphasise specific anatomical regions such as the sacrum, retrosacral space, presacral fascia, and coccyx [11, 12]. In this study, PPE was defined as extraction of the internal reproductive organs (uterus, cervix, and vagina) and rectum while preserving the bladder [13]. R_0_ resection was defined as tumour resection with pathologically confirmed negative margins, devoid of distant metastasis, and maintaining a CRM exceeding 1 mm. Surgical interventions were exclusively performed by surgeons with more than 10 years of experience in rectal cancer resection. Based on our experience with pelvic exenteration, we introduced an innovative Peking classification system distinct from the PPE infralevator (PPEI) and PPE supralevator (PPES). Within this classification, PPE was categorised into four subtypes based on the preoperative MRI results and the specific processes involved in the surgery: PPE-I (uterine invasion), PPE-II (upper vaginal invasion), PPE-III (lower vaginal invasion), and PPE-IV (major vaginal invasion). PPE-I involved instances where the uterus faced upper uterine invasion, prompting an anterior resection (AR) combined with a hysterectomy. The vagina was wholly preserved (Fig. 1a), with subsequent suturing and performance of colorectal anastomosis or Hartmann’s procedure [14, 15]. PPE-II encompassed cases of middle or low rectal cancer infiltration into the upper vagina or cervix, warranting low anterior resection (LAR) and hysterectomy coupled with upper vaginal dissection. Partial vaginal preservation and suturing followed this procedure (Fig. 1b). PPE-III was determined when the lower vagina encountered invasion, necessitating abdominal perineal resection (APR) and posterior vaginal wall wedge resection, followed by suturing (Fig. 1c). PPE-IV corresponded to instances mirroring PPE-II during the abdominal phase while requiring complete dissection of the posterior wall. Reconstruction of the residual vagina was undertaken via V-Y plasty or gluteal fold flap reconstruction (Fig. 1d).

### Statistical analysis

Statistical analyses were carried out using IBM SPSS Statistics 26 (IBM, Inc., Armonk, NY, USA). Categorial variable comparisons were undertaken through Pearson’s chi-squared and Fisher’s exact tests, while continuous variables were analysed using the Kruskal–Wallis H test. To ascertain probabilities associated with OS, DFS, and LRS, the Kaplan–Meier method was employed, with subsequent comparisons using the log-rank test to identify significant differences in outcomes. Variables found to be significant in the univariate analysis were further examined using a Cox regression model in a multivariate analysis. *P*-values < 0.05 were considered statistically significant.

## Results

### Patient characteristics

**Fig. 2:**
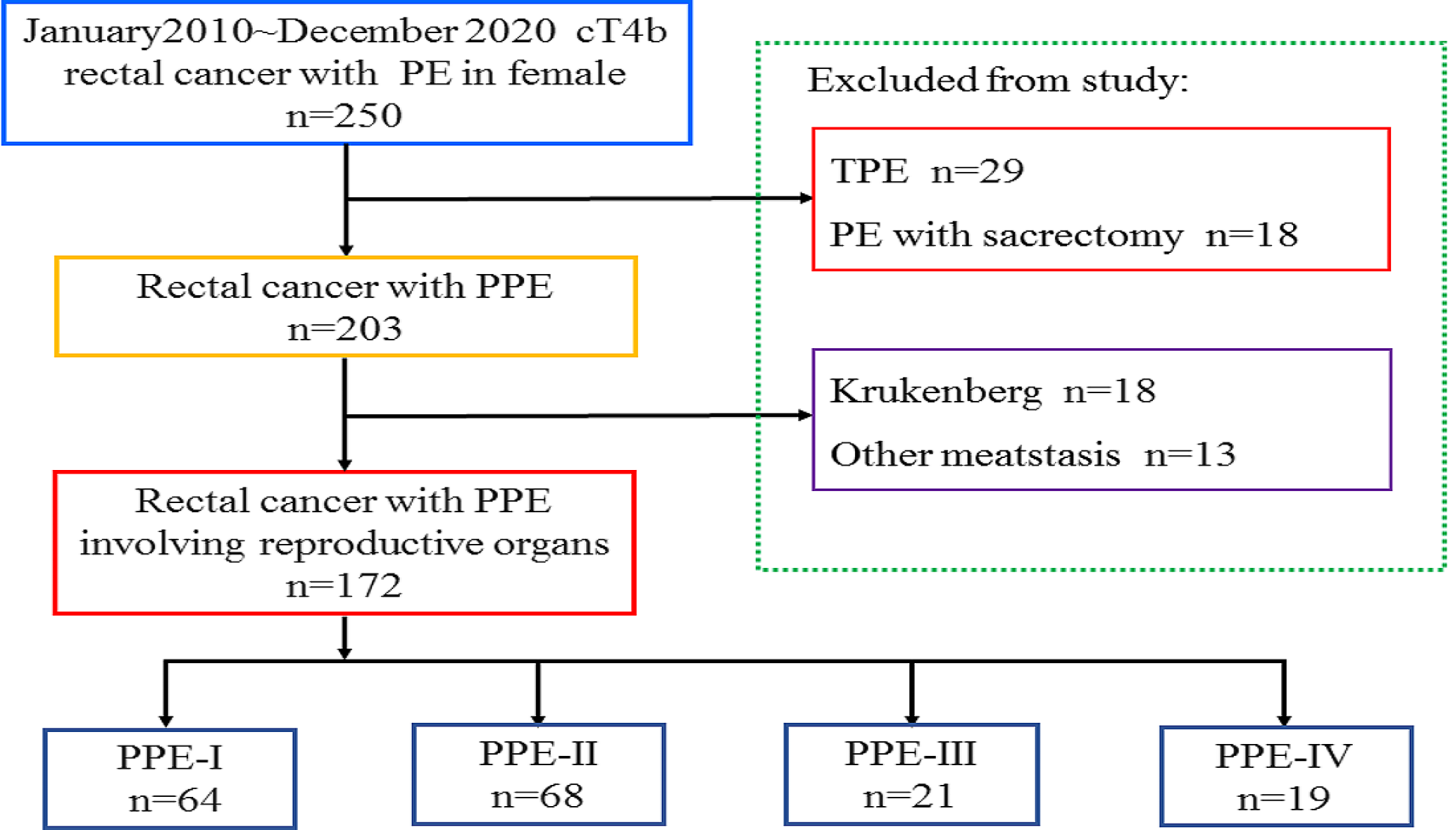
Flowchart of the patient selection strategy

A total of 172 patients who had been diagnosed with rectal cancer and underwent PPE involving the inner reproductive organs were included in this study, and they were separated into four distinct groups: PPE-I 37.2% (64/172), PPE-II 39.6% (68/172), PPE-III 12.2% (21/172), and PPE-IV 11.0% (19/172). The study’s flowchart is depicted in Fig. 2, while a comprehensive overview of baseline patient characteristics is provided in Table 1. Across the four groups, no statistically significant differences were observed in age, BMI, ASA score, comorbidities, neoadjuvant therapy, surgical approach, tumour size, tumour differentiation, (y)pT4b stage, (y)pN + stage, history of abdominal surgery, or laboratory test results between the groups.

**Table 1:** Characteristics of patients in four groups

Variables	PPE-I (64)	PPE-II (68)	PPE-III (21)	PPE-IV (19)	*P*
Age > 60, years	32(50.0)	33(48.5)	11(52.4)	10(52.6)	0.98
BMI > 23.9, kg/m^2^	28(43.8)	25(36.8)	9(42.9)	8(42.1)	0.86
ASA category					0.97
I-II	56(87.5)	59(86.8)	19(90.5)	17(89.5)	
III	8(12.5)	9(13.2)	2(9.5)	2(10.5)	
Comorbidity	30(46.9)	31(45.6)	7(33.3)	12(63.2)	0.31
Surgery history	17(26.6)	22(32.4)	5(23.8)	8(42.1)	0.53
Preoperative treatment					0.70
Chemoradiotherapy	19(29.7)	17(25.0)	8(38.1)	6(31.6)	
None	45(70.3)	51(75.0)	13(61.9)	13(68.4)	
Surgical approach					0.35
Open	36(56.3)	28(41.2)	10(47.6)	8(42.1)	
Laparoscopic	28(43.7)	40(58.8)	11(52.4)	11(57.9)	
Tumor size, mm					0.48
>35	54(84.4)	53(77.9)	17(81.0)	13(68.4)	
≤ 35	10(15.6)	15(22.1)	4(19.0)	6(31.6)	
Differentiation degree					0.79
Well/ Moderate	45(70.3)	38(55.9)	14(66.7)	11(57.9)	
Poor	19(29.7)	30(44.1)	7(33.3)	8(42.1)	
(y)pT stage					0.11
T_0_-T_4a_	32(50.0)	23(33.8)	5(23.8)	8(42.1)	
T_4b_	32(50.0)	45(66.2)	16(76.2)	11(57.9)	
(y)pN stage					0.72
N_0_	37(57.8)	33(48.5)	11(52.4)	9(47.4)	
N_1_-N_2_	27(42.2)	35(51.5)	10(47.6)	10(52.6)	
Vascular invasion	18(28.1)	18(26.5)	5(23.8)	6(31.6)	0.95
Perineural invasion	13(20.3)	20(29.4)	6(28.6)	7(36.8)	0.45
Adjuvant therapy	44(68.8)	54(79.4)	14(66.7)	15(78.9)	0.43
Hb < 110 g/L	28(43.8)	20(29.4)	9(42.9)	6(31.6)	0.32
ALB < 35 g/L	16(25.0)	17(25.0)	4(19.0)	2(10.5)	0.54
CEA > 5 ng/ml	28(43.8)	35(51.5)	8(38.1)	8(42.1)	0.66
CA199 > 37 U/ml	9(14.1)	15(22.1)	4(19.0)	6(31.6)	0.40
Enrolled centers					
NCC	35(54.7)	40(58.8)	7(33.3)	8(42.1)	0.11
PKUFM	24(37.5)	17(25.0)	10(47.6)	10(52.6)	
GSH	5(7.8)	11(16.2)	4(19.1)	1(5.3)	

*Abbreviations*: BMI: body mass index; ASA: American Society of Anesthesiologists; nCRT: neoadjuvant chemotherapy; PPE: posterior pelvic exenteration; (y)pT4b/N+: pathologic T4b stage or having positive lymph nodes retrieved with or without neoadjuvant therapy; CEA: carcinoembryonic antigen; CA199: carbohydrate antigen 199; NCC: National Cancer Center; PKUFM: Peking University First Hospital; GSH: Gansu Provincial Hospital

### Perioperative and pathological results

The intraoperative data and pathological results are presented in Table 2. Significant differences were observed between the four groups in terms of the type of colorectal reconstruction (*p* < 0.001) and perineal reconstruction (*p* < 0.001). However, no significant differences were identified in the operative time, blood loss, or the count of retrieved and positive lymph nodes between the groups. The R_0_ resection rates of the PPE-I, PPE-II, PPE-III, and PPE-IV subgroups were 90.6%, 89.7%, 90.5%, and 89.5%, respectively, with an overall positive CRM rate of 9.9%.

**Table 2:** Intraoperative and pathologic characteristics in four groups

Variables	PPE-I (64)	PPE-II (68)	PPE-III (21)	PPE-IV (19)	*P*
Operative time, min	266(212–356)	240(203–297)	255(192–308)	253(205–357)	0.21
blood loss, ml	265(100–500)	100(50–315)	100.0(50–725)	200(60–500)	0.11
Colorectal reconstruction					< 0.001
LAR	55(85.9)	48(70.6)	0(0)	0(0)	
Hartmann	9(14.1)	20(29.4)	0(0)	0(0)	
APR	0(0)	0(0)	21(100)	19(100)	
Perineal reconstruction					< 0.001
Suture	64(100)	68(100)	21(100)	0(0)	
Plasty	0(0)	0(0)	0(0)	19(100)	
No. of retrieved lymph nodes	22(15–29)	21(14–27)	20(14–24)	16(13–24)	0.32
No. of positive lymph nodes	0(0–3.0)	0.5(0–2.0)	1(0–2.0)	1(0–2.0)	0.998
CRM status					0.998
CRM > 1 mm	58(90.6)	61(89.7)	19(90.5)	17(89.5)	
CRM ≤ 1 mm	6(9.4)	7(10.3)	2(9.5)	2(10.5)	

*Abbreviations*: APR: abdominal perineal resection; CRM: circumferential resection margin

### Postoperative recovery and complications

Information on postoperative recovery and complications is shown in Table 3. There were no reports of postoperative deaths in any of the groups. The rate of complications that occurred while patients were in the hospital was 36.6% (63/172 patients). The complication rates for PPE-I, PPE-II, PPE-III, and PPE-IV were 32.8%, 33.8%, 47.6%, and 47.4%, respectively (*p* < 0.001). Notably, no significant differences were detected between the four groups in reoperation rate (*p* = 0.49), length of postoperative hospitalisation (*p* = 0.69), anastomotic leakage rate (*p* = 0.84), and the surgical site infection (SSI) complication rate, including abdominal incisions (*p* = 0.60), perineal incisions (*p* = 0.16), pelvic infections (*p* = 0.37), and urinary infections (*p* = 0.09). Compared to that of the PPE-III/IV group, the PPE-I/II group had a significantly lower rate of urinary retention (*p* = 0.04).

**Table 3:** Postoperative parameters among four groups

Variables	PPE-I (64)	PPE-II(68)	PPE-III (21)	PPE-IV (19)	*P*
Mortality	0(0)	0(0)	0(0)	0(0)	1.00
Clavien–Dindo grade					0.03
0	43(67.2)	45(66.2)	11(52.4)	10(52.6)	
I-II	19(29.7)	21(30.9)	6(28.6)	5(26.3)	
III-IV	2(3.1)	2(2.9)	4(19.0)	4(21.1)	
Surgical reintervention	2(3.1)	2(2.9)	1(4.8)	2(10.5)	0.49
Surgical site infection	13(20.3)	8(11.8)	2(9.5)	5(26.3)	0.27
Abdominal incision	7(10.9)	5(7.4)	1(4.8)	3(15.8)	0.60
Perineal incision^a^	-	-	2(9.5)	5(26.3)	0.16
Pelvic infection	5(7.8)	4(5.9)	0(0)	0(0)	0.37
Urinary infection	1(1.6)	1(1.5)	0(0)	2(10.5)	0.09
Rectal anastomotic leakage^b^	4(7.3)	3(6.3)	-	-	0.84
Intestinal leakage/fistula	0(0)	0(0)	0(0)	2(10.5)	0.001
Urinary leakage	1(1.6)	0(0)	0(0)	0(0)	0.64
Ileus	4(6.3)	7(10.3)	2(9.5)	1(5.3)	0.80
Pulmonary embolism	0(0)	1(1.5)	0(0)	0(0)	0.67
Urinary retention	6(9.4)	5(7.4)	6(28.6)	4(21.1)	0.04
Length of stay (days)	11(9–16)	11(8–14)	12(8–14)	10(8–15)	0.69

a: For patients receiving abdominal perineal resection only (40 cases in PPE-III and PPE-IV group)

b: For patients receiving colorectal anastomosis only (132 cases in PPE-I and PPE-II group)

### Oncological outcomes

**Fig. 3:**
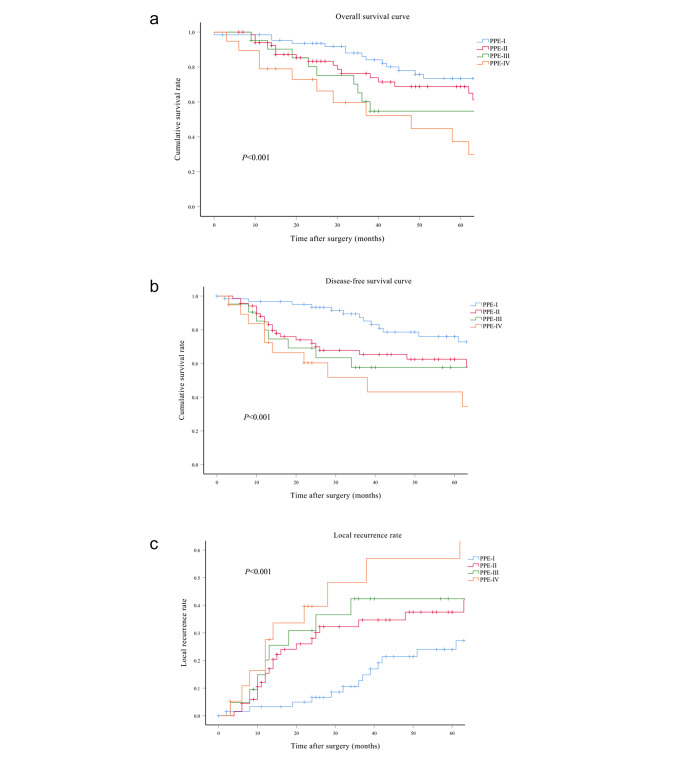
Kaplan–Meier survival curves comparing overall survival (**a**), disease-free survival (**b**), and local recurrence (**c**) for different types of PPE classified by the new Peking classification

**Fig. 4:**
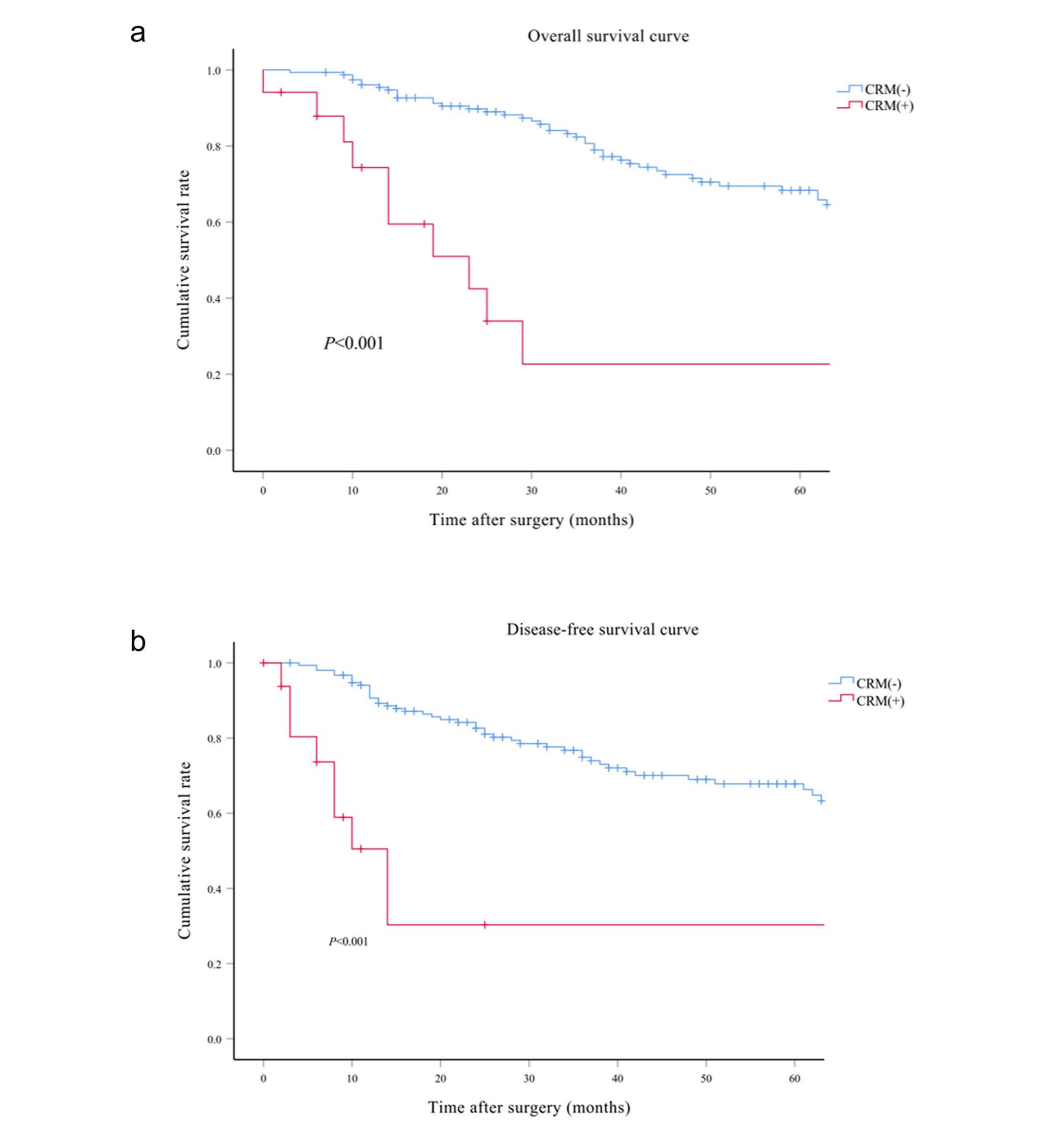
Kaplan–Meier survival curves comparing overall survival (**a**) and disease-free survival (**b**) in the CRM (+) and CRM (–) groups

**Fig. 5:**
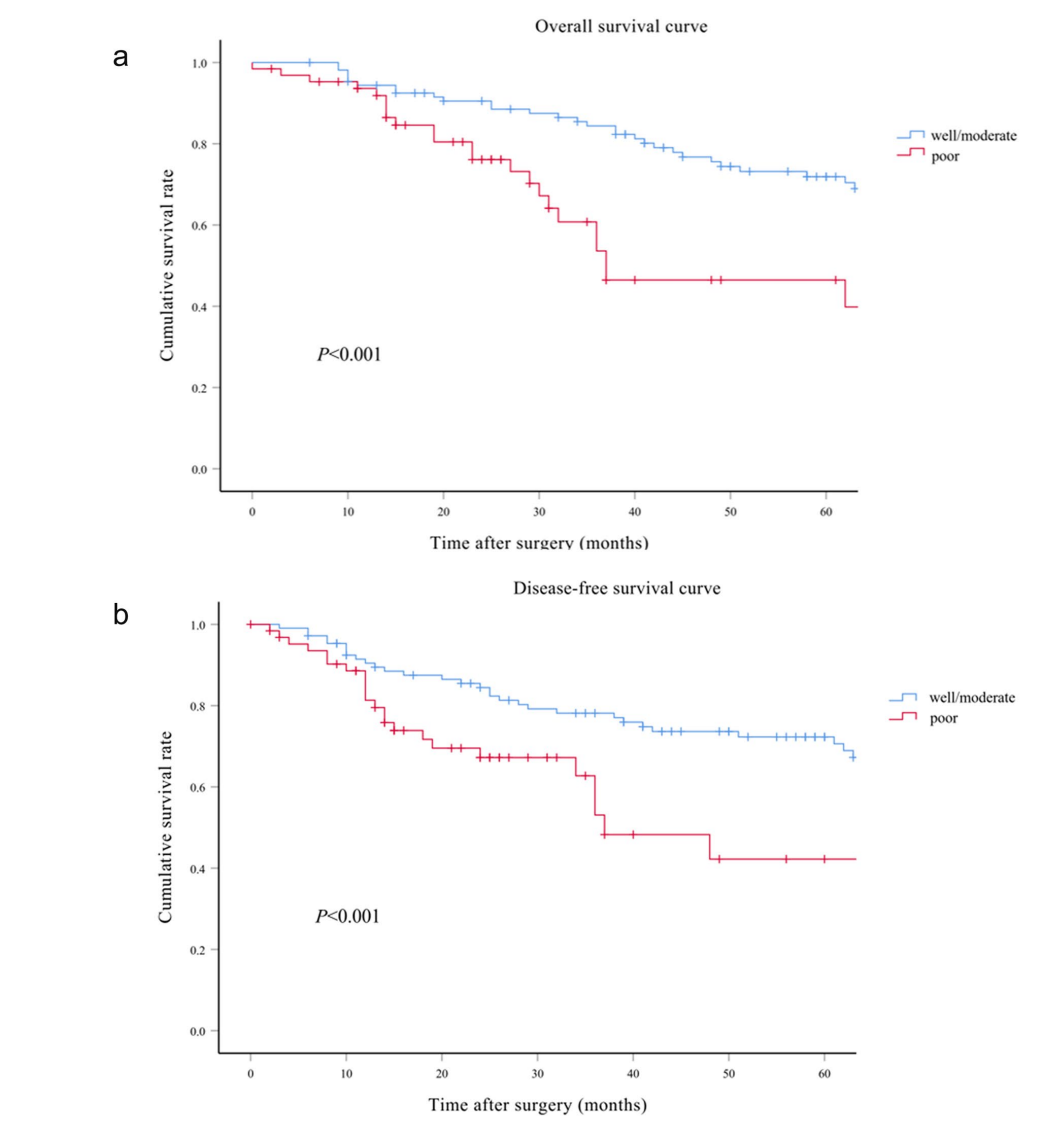
Kaplan–Meier survival curves comparing overall survival (**a**) and disease-free survival (**b**) in the well/moderate differentiation and poor differentiation groups

The median follow-up period spanned 40 months (interquartile range: 21–69 months) for the entire cohort, with subsequent subdivisions into 51 months (interquartile range: 32–89 months) for the PPE-I group, 31 months (interquartile range: 15–63 months) for the PPE-II group, 38 months (interquartile range: 24–79 months) for the PPE-III group, and 29 months (interquartile range: 13–62 months) for the PPE-IV group. Comprehensive follow-up was successfully conducted for all patients throughout the study period. The four groups showed significant differences in OS (*p* < 0.001), DFS, and LRS (*p* < 0.001) (Fig. 3a and b, and 3c). The 5-year OS rates were 73.4%, 68.8%, 54.7%, and 37.3% for the PPE-I, PPE-II, PPE-III, and PPE-IV groups, respectively. Correspondingly, the 5-year DFS rates for the PPE-I, PPE-II, PPE-III, and PPE-IV groups were 76.0%, 62.5%, 57.7%, and 43.1%, respectively, whilst the 5-year LRS rates were 24.0%, 37.5%, 42.3%, and 56.9%. Additionally, there were significant differences in the 5-year OS and DFS rates among the subgroups categorised by CRM status (Fig. 4a and b) (*p* < 0.001) and tumour differentiation (Fig. 5a and b) (*p* < 0.001). The 5-year OS rates reached 68.3% and 22.7% for the negative and positive CRM groups, respectively. Meanwhile, the 5-year DFS rates were 67.8% and 30.3% for the same subgroups. In terms of tumour differentiation, the 5-year OS rates amounted to 71.9% and 46.5% for well/moderately and poorly differentiated subgroups, respectively. Similarly, the 5-year DFS rates were 72.3% and 42.2% for the same subgroups.

The influence of prognostic factors on OS and DFS, as determined by univariate and multivariate analyses, is presented in Table 4. In the univariate analysis, age > 60 years, higher Peking classification type, positive CRM, (y)pT stage, and poor differentiation were identified as independent risk factors for both OS and DFS (*p* < 0.05). Further analysis revealed that a higher Peking classification type (hazard ratio [HR] = 3.48, 95% confidence interval [CI], 1.65–7.36, *p* < 0.001), negative CRM status (HR = 3.59, 95% CI, 1.80–7.17, *p* < 0.001), and poor differentiation (HR = 2.81, 95% CI, 1.64–4.83, *p* < 0.001) independently contributed to OS. In parallel, a higher Peking classification type (HR = 4.12, 95% CI, 1.85–9.15, *p* < 0.001), negative CRM status (HR = 2.89, 95% CI, 1.23–6.83, *p* = 0.015), and poor differentiation (HR = 2.12, 95% CI, 1.27–3.81, *p* = 0.005) were established as independent risk factors for DFS.

**Table 4:** Univariate and multivariate analysis for overall survival and disease-free survival

Variables	Overall survival				Diseases-free survival			
	Univariate analysis		Multivariate analysis		Univariate analysis		Multivariate analysis	
	HR (95%CI)	*p*	HR (95%CI)	*p*	HR (95%CI)	*p*	HR (95%CI)	*p*
Age	1.68(1.06–2.07)	0.03	1.53(0.95–2.47)	0.08	1.56(0.96–2.55)	0.07	1.49(0.87–2.54)	0.15
nCRT (yes/no)	0.82(0.50–1.33)	0.42			1.00(0.60–1.65)	0.99		
CEA	1.28(0.81–2.02)	0.30			1.24(0.76–2.01)	0.40		
CA199	1.21(0.70–2.11)	0.49			1.14(0.63–2.05)	0.67		
Surgical approach (laparoscopic/open)	0.80(0.51–1.28)	0.35			0.67(0.41–1.11)	0.12		
PPE classification (PPE-I)		< 0.001		0.01		< 0.001		0.005
PPE-II	2.58(1.41–4.72)	0.002	1.92(1.03–3.60)	0.04	3.15(1.64–6.03)	0.001	2.46(1.27–4.78)	0.008
PPE-III	2.57(1.26–5.25)	0.010	2.20(1.05–4.60)	0.04	2.71(1.17–6.26)	0.02	2.47(1.06–5.78)	0.04
PPE-IV	5.84(2.92–12.10)	< 0.001	3.48(1.65–7.36)	0.001	6.50(3.03–13.90)	< 0.001	4.12(1.85–9.15)	0.001
CRM status (positive/negative)	4.27(2.22–8.20)	< 0.001	3.59(1.80–7.17)	< 0.001	4.41(2.23–8.74)	< 0.001	2.89(1.23–6.83)	0.015
Perineural invasion (yes/no)	1.25(0.72–2.15)	0.43			1.72(1.01–2.92)	0.045	1.22(0.62–2.42)	0.56
Lymphatic invasion (yes/no)	0.67(0.40–1.24)	0.20			0.91(0.50–1.64)	0.74		
Differentiation (well/ moderate, poor)	3.45(2.03–5.87)	< 0.001	2.81(1.64–4.83)	< 0.001	2.65(1.54–4.56)	< 0.001	2.12(1.27–3.81)	0.005
(y)pT stage(T4b/non-T4b)	1.67(1.03–2.72)	0.04	1.03(0.61–1.73)	0.91	1.87(1.10–3.21)	0.02	1.18(0.67–2.07)	0.57
(y)pN stage (N1-2/N0)	1.17(0.74–1.84)	0.51			1.39(0.86–2.27)	0.18		
Postoperative complications(yes/no)	1.31(0.83–2.08)	0.24			1.47(0.91–2.38)	0.12		
Adjuvant therapy(yes/no)	1.30(0.74–2.25)	0.37			1.89(0.75–4.83)	0.17		

*Abbreviations*: CEA: carcinoembryonic antigen; PPE: posterior pelvic exenteration; CRM: circumferential resection margin

## Discussion

Currently, there is a lack of both surgical and oncological evidence to support the use of PPE in cases of cT4b rectal cancer involving the female internal reproductive organs. This study stands as the largest and first investigation of cT4b rectal cancer involving the female internal reproductive organs. Based on this new classification, our findings demonstrate the safety and feasibility of PPE involving the female internal reproductive organs.

Traditionally, PPE is classified into PPEI and PPES based on rectal cancer height and anal preservation. Some individuals argue that partial vaginal resection should not be considered part of PPE, while others believe it can still be included. Indeed, there is controversy surrounding this issue. Our surgical experience led to the formulation of the novel Peking classification for PPE, closely associated with the surgical plan. This classification considers both the site of reproductive system invasion and rectal cancer height. Notably, the results of this study indicate a significant correlation between oncological outcomes, including OS and DFS, and the Peking classification grade. Higher grades of classification are indicative of poorer oncological outcomes. The results indicate that further studies into PPE-IV management, encompassing preoperative multidisciplinary consultation, neoadjuvant chemoradiotherapy, and extended radical excision, are warranted to enhance surgical and oncological outcomes.

A study conducted by the National Cancer Center of Mexico enrolled 59 patients, with 51 and 8 undergoing PPE and TPE, respectively. The study reported an operative mortality rate of 3% (two cases), while postoperative complications were experienced by 29 patients (49.2%) [16]. Our study’s overall in-hospital complication rate was 36.6% (63/172). Between the four groups, there were no statistically significant differences in terms of reoperation rate (*p* = 0.49), postoperative hospital stay (*p* = 0.69), anastomotic leakage rate (*p* = 0.84), or SSI complication rate (*p* = 0.27). Our study’s incidence of complications was lower than that reported in other studies [17, 18], a trend potentially linked to the evolution of laparoscopy in PPE surgery and surgical approaches based on the Peking classification. Prognostic factors linked to survival rate in patients undergoing PPE include pathological nodal status, preoperative radiotherapy and chemotherapy, and pathological node status, although there is a lack of consensus. In our study, multivariate analysis identified a higher Peking classification type, CRM status, and poor differentiation as independent risk factors for both OS and DFS (*p* < 0.001). This underscores the significance of our proposed PPE classification for cT4b rectal cancer involving the female internal reproductive organs.

The R_0_ resection rate stands as a critical parameter for assessing surgical quality. Unlike TPE, PPE allows organ-sparing surgery, preserving the bladder and innervating nerves. Consequently, achieving R_0_ resection is more challenging in PPE than in TPE. To preserve the bladder and pelvic plexus nerves during PPE, the dissection plane must be close to the parametrial tissue and paracolpium. This factor can impact the attainment of R_0_ resection [19, 20]. In our study, the R_0_ resection rates for the PPE-I, PPE-II, PPE-III, and PPE-IV subgroups were 90.6%, 89.7%, 90.5%, and 89.5%, respectively. The 5-year OS and DFS rates in the negative CRM subgroup were 68.3% and 67.8%, respectively. A recent systematic review reported a low median R_0_ resection rate of 82.6% (range 66–95.5%) for LARC and a low median 5-year survival rate of 32% [21]. Our results show promise, potentially attributed to the focused inclusion of patients with rectal cancer involving the female internal reproductive organs. Furthermore, adopting this new PPE classification, which may facilitate varied surgical approaches based on tumour location, might improve R_0_ resection rates and enhance long-term survival outcomes.

This study has some limitations, such as its retrospective design, potential patient selection bias, and the relatively small number of cases. Additionally, the results may have been influenced by the limited data available for evaluating bladder function. Further research is needed to determine if the findings are applicable to male patients. Finally, less than one-third of the patients with LARC received nCRT, and the lack of clinical data on postoperative adjuvant therapy may also affect the prognosis by introducing bias into the analysis of the effect of the neoadjuvant on survival.

## Conclusions

This study introduced a novel classification of PPE; to the best of our knowledge, it is the most extensive assessment of short-term outcomes and oncological results in female patients to date. The new Peking classification facilitates precise surgery and its grades correlate with survival outcomes. Investigating the impact of inflammation and cancer invasion in different reproductive organs on prognosis represents a promising avenue for future research. Therefore, further studies on the management of PPE-IV, in terms of preoperative multidisciplinary team consultation, neoadjuvant chemoradiotherapy, and extended radical excision, should be performed to improve surgical and oncological outcomes.

## Electronic supplementary material

Below is the link to the electronic supplementary material.

Supplementary Material 1

## Data Availability

No datasets were generated or analysed during the current study.
